# Lycorine inhibits migration and proliferation of hepatocellular carcinoma cells by reducing transketonase expression

**DOI:** 10.7150/jca.93026

**Published:** 2024-02-04

**Authors:** Li-Li Ge, Hui Wang, Yi-Hong Zhang, Hui-Ju Wang, Li Li

**Affiliations:** 1 Department of Nursing,Zhejiang Provincial People's Hospital (Affiliated People's Hospital), Hangzhou Medical College, Hangzhou, Zhejiang, China.; 2 Department of Ultrasound, Taian Maternal and Child Health Hospital, Taian 310014, Shandong Province, China.; 3 General Surgery, Cancer Center, Department of Gastrointestinal and Pancreatic Surgery, Zhejiang Provincial People's Hospital (Affiliated People's Hospital), Hangzhou Medical College, Hangzhou, Zhejiang, China.

**Keywords:** Lycorine, Proliferation, Migration, Hepatocellular carcinoma cells, Transketolase

## Abstract

**Background:** Previous studies have showed that lycorine can restrain the development of multiple tumor types, containing hepatocellular carcinoma (HCC), but the underlying mechanisms remain unknown.

**Methods:** We assessed the impact of lycorine on hepatocellular cancer cell proliferation, migration, colony formation, cell cycle, and apoptosis. The possible inhibitory effect of lycorine on the activity of HCC cells was analyzed by RNA-seq, and transketolase (*TKT*) expression in HCC and nontumorous tissues was detected using RT-PCR. The expression of TKT protein in HCC and tumor adjacent non-cancerous tissues was detected by immunohistochemistry. We evaluated the association of expression of TKT in HCC tissues with prognosis, and investigated the inhibitory effect of lycorine on tumor growth in vivo.

**Results:** Lycorine significantly inhibited the proliferation, invasion, migration, colony formation, cell cycle of HCC cells, but had no obvious impact on apoptosis. Twenty-eight genes were found to be down-regulated in HuH7 and HepG2 cells after lycorine treatment, and the difference of TKT gene expression was significantly. The expression of TKT protein was significantly higher in HCC than in non-tumorous tissues. The expression of TKT was correlated with tumor size, Edmondson grade, AFP, and overall survival. Survival analysis suggested that high expression of TKT was associated with a poor survival. The average tumor volume and weight were significantly reduced in the lycorine injection group, but the body weights of the mice did not change significantly.

**Conclusion:** Lycorine can restrict the migration and proliferation of HCC cells by down-regulating TKT expression, and it may be a potential meaningful drug for the prevention and treatment of HCC.

## Background

Hepatocellular carcinoma (HCC) is a common digestive system tumor characterized by high malignancy, rapid development, and high mortality. China has a large number of liver cancer cases, contributing to about 50% of the total number of cases and deaths worldwide^1^. Currently, surgery remains as the most effective treatment for HCC. However, HCC has an insidious onset and lacks typical clinical symptoms at the early stage. Diagnosis is mostly made at the middle and late stages, and only about 15% of HCC patients can be treated by surgical resection^2^.

Lycorine is an isoquinoline alkaloid extracted from the bulbs of Amaryllidaceae plants. It possesses many biological effects, such as anti-viral, anti-inflammatory, and anti-malarial activities, protects the cardiovascular system, and has significant anti-cancer effects against various tumor cell types^3^-^6^. It is reported that lycorine hydrochloride induces apoptosis and reduces cell proliferation by promoting the FBXW7-MCL1 axis in gastric cancer^7^, suppresses tumor growth of human osteosarcoma cells by blocking the Wnt/β-catenin, PI3K/AKT, and ERK1/2/MAPK signaling pathways^8^, and induces colorectal cancer cell apoptosis by regulating MEK2 and enhances vemurafenib activity^9^.

Transketolase (TKT) is a metabolic enzyme participating in the non-oxidative branch of the pentose phosphate pathway (PPP) and links PPP and glycolysis. Studies have found that TKT was related with tumor metastasis and a poor survival in breast cancer and non-small cell lung cancer^10^,^11^. Abnormal expression of TKT has been found in HCC. TKT can translocate into the nucleus of HCC cell lines, interact with signal transducer and activator of STAT1, and inhibit FXR expression by accelerating the binding between HDAC3 and *FXR* promoter^12^. Lycorine accelerates autophagy and apoptosis through TCRP1/Akt/mTOR pathway in HCC^13^.

This study further explored whether lycorine inhibits the invasion and migration of HCC cells by downregulating TKT as well as the underlying mechanism.

## Materials and methods

### Clinical samples

A total of 200 HCC specimens were collected at Zhejiang Provincial People's Hospital from January 2010 to January 2018. One hundred and forty cases were smaller than 5 cm and 60 cases were larger than 5 cm; 136 cases were Edmondson grades I-II and 64 were grade III (Table 1). The study was approved by the Ethics Committee of Zhejiang Provincial People's Hospital (The approved number:QT2022295). All participants signed an informed consent form. And the study was conducted in accordance with the Declaration of Helsinki.

### Cell lines

The human HCC cell lines HepG2 and Huh7 were offered by the Key Laboratory of Gastroenterology of Zhejiang Province and were cultured in DMEM (Gibco, Beijing, China) containing 10% fetal bovine serum (Gibco, Beijing, China), 50 U/mL penicillin, and 50 μg/mL streptomycin. Cells were maintained at 37 °C with 5% CO_2_.

### MTS assay

HepG2 and Huh7 cells were seeded in 96-well plates at a density of 500 cells/well, incubated overnight, and treated with lycorine (Enzo Life Sciences, Inc, United States) at various concentrations (0, 0.01, 0.10, 1.00, 2.00, 5.00, 20.00, 50.00, 100.00, and 200.00 μM) for 24, 48 h and 72h. Then, 50 μL of PMS was added to 1 mL of MTS, and 20 μL of the mixture was added to each well. Finally, OD values were read at 490 nm after incubating with MTS for 4 h. Cell growth was observed continuously for 3 d, and cell growth curves were plotted to calculate IC_50_ concentration with SPSS software.

### Colony formation assay

HepG2 and Huh7 cells were seeded in 6-well plates (500 cells per well) and cultured overnight. Then, the cells were treated with lycorine at various concentrations (0, 0.2, and 2.0 μM) for 3 weeks, fixed with absolute ethanol, stained with crystal violet, and photographed to count the number of colonies. Cell colonies were washed with phosphate buffered saline (PBS), fixed with absolute ethanol for 15 min, and stained with crystal violet for 10 min. Photographs were subsequently taken and only colonies containing more than 50 cells were recorded.

### Migration assay

HepG2 and Huh7 cells(2×10^5^) were seeded in the upper chambers of transwell inserts and treated with various concentrations of lycorine (0 μM and 0.2 μM) for 24 h. The cells on the membrane were subjected to methanol fixation and Giemsa staining, and then counted under a microscope.

### Cell cycle analysis

HepG2 and Huh7 cells in the logarithmic growth phase were seeded in 6-well plates (1×10^5^ cells per well) and cultured overnight. Lycorine (0 μM and 2 μM) was applied to the cells for 48 h. The cells were then collected, washed with PBS, fixed with 75% ethanol for 4 h, washed again with PBS, and then incubated with cell cycle dye. The cell cycle was then analyzed using flow cytometry.

### Cell apoptosis analysis

HepG2 and Huh7 cells in the logarithmic growth phase were seeded in 6-well plates (1 × 10^5^ cells per well) and cultured overnight. Lycorine (0 μM and 2 μM) was applied to the cells for 48 h. The cells were collected, washed twice with the precooled cell standing buffer, and resuspended with the Amexin V binding buffer. Then, 100 uL of cell suspension was transferred to a 5 mL reaction tube, and 5 μL of FITC Amexin V and 10 μL of PI solutions were added. After incubation at room temperature in dark for 15 min, 400 uL of Amexin V binding buffer was added. Cell apoptosis was then analyzed using flow cytometry.

### RNA-seq

HepG2 and Huh7 cells were cultured in DMEM medium containing 10% fetal bovine serum in an incubator with 5%CO_2_ incubator at 37°C. When the cell density reached about 70%, lycorine was added at a concentration of 4 μM. After 48 h, the cells were collected and stored at -80°C with 1 mL Trizol (Invitrogen, United States). Cells were divided into two groups according to whether lycorine was added or not, and transcriptome analysis was performed by Huada Gene (Beijing, China). We used on-line Venn map (https://bioinfogp.cnb.csic.es/tools/venny/index.html) to screen out the genes that were downregulated in both cell lines after adding lycorine^14^.

### RT-qPCR

HepG2 cells in the logarithmic growth phase were seeded in 6-well plates (1 × 10^5^ cells per well) and cultured overnight. Lycorine (2 μM) was applied to the cells for 48 h. Trizol was used to extract total RNA and reverse transcription was performed to synthesize cDNA. The primers used for *TKT* amplification are: Forward, 5'-TGTGTCCAGTGCAGTAGTGG-3' and reverse, 5'-ACACTTCATACCCGCCCTAG-3'. qPCR was performed according to the manufacturer's instructions as previously described^15^.

### Western blot analysis

Whole cell protein lysates were extracted and protein concentrations then quantified using a BCA protein assay kit (Pierce, Rockford, IL, United States). Western blotting was performed according to standard procedures. Transketolase antibody (TKT, 1:2500) used was from GeneTex (United States), and β-actin (HUABIO, China, 1:5000) served as the loading control.

### Animal experiments in vivo

All animal experiments were conducted strictly in accordance with the protocol approved by the Institutional Animal Care and Use Committee of Zhejiang Provincial People's Hospital. The specific operation was as follows: HepG2 cells (5×10^6^) were injected into 5-week-old BALB/c female mice via subcutaneous injection in 0.2mL PBS. When the tumor volume reached 100 mm3, the mice were randomly divided into 2 groups (n=5 per group). One group was injected with lycorine (10mg/kg/day) every other day for 14 days, while another group received the same volume of PBS. The tumor volume and body weight of each mouse were monitored and recorded every 3 days throughout the experiment. Finally, after the experiment was completed, the mice were sacrificed, and their tumors were weighed, photographed.

### Database analysis

*TKT* expression in hepatocellular carcinoma and normal tissues was compared based on the Oncomine (https://www.oncomine.org), UALCAN (http://ualcan.path.uab.edu/analysis.html), and GEPIA database (http://gepia.cancer-pku.cn/index.html) databases^16^,^17^.

The association of TKT expression with survival in HCC was analyzed with the Kaplan-Meier plotter (http://kmplot.com/analysis/) based on the GEPIA database^18^,^19^. Hazard ratios (HRs) with 95% confidence intervals (CIs) and log-rank *P*-values were also computed. We analyzed TKT centered gene network and protein-protein interaction network using Gene MANIA (http://genemania.org/) and STRING (https://string-db.org/) online tools^20^,^21^.

### Immunohistochemistry

Immunohistochemistry was conducted using a rabbit anti-TKT antibody (1:500; GeneTex, North America), according to the manufacturer's instructions. TKT expression appeared as brown granular spots, mainly localized in the cytoplasm and membrane of tumor cells. TKT expression was judged according to a previous report^22^.

### Statistical analysis

SPSS 19 statistical software was used for statistical analyses. Enumeration data were analyzed using *χ*^2^ or Fisher exact probability tests. Measurement data were analyzed using the *t*-test. Survival data were assessed using Kaplan-Meier analysis. Survival curves were plotted and differences were analyzed using the log-rank test.

## Results

### Lycorine significantly inhibits HCC cell proliferation and migration

**Figure 1:**
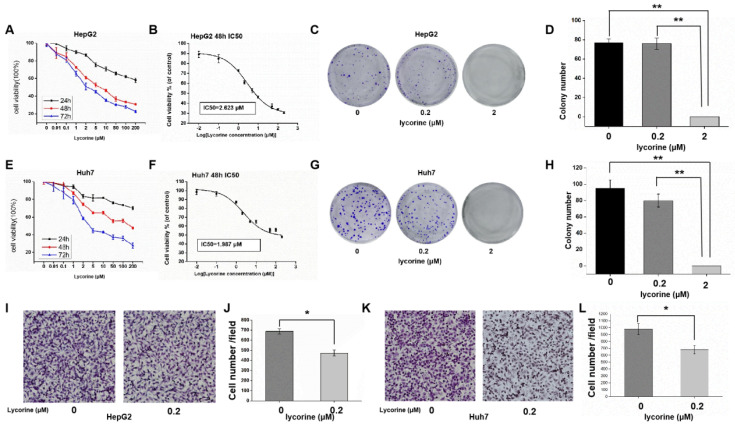
** Lycorine significantly inhibits the proliferation and migration of hepatocellular carcinoma cells.** A: MTS assay showing that the relative activity of HepG2 cells was decreased gradually with the increase in lycorine concentration and time; B: IC50 for HepG2 cells at 48 h was 2.623 uM; C and D: Lycorine effectively inhibits the colony-forming ability of HepG2 cells (**P<0.01); E: MTS assay showing that the relative activity of Huh7 cells was decreased gradually with the increase in lycorine concentration and time; F: IC50 for Huh7 cells at 48 h was 1.987 μM; G and H: Lycorine effectively inhibits the colony-forming ability of Huh7 cells (**P<0.01); I and J: Lycorine effectively inhibits the migratory ability of HepG2 cells (*P<0.05); K and L: Lycorine effectively inhibits the migratory ability of Huh7 cells (*P<0.05).

MTS results showed that the relative activity of HCC cells decreased gradually with the increase of lycorine concentration and incubation time (Figure 1A and E). Using SPSS software to calculate the IC_50_ concentration, it was found that the IC_50_ concentration was 2.623 μM for HepG2 cells (Figure 1B) and 1.987 μM for Huh7 cells (Figure 1F), indicating that a low concentration of lycorine significantly inhibited the proliferation of HuH7 and HepG2. Colony formation assays revealed that in Huh7 and HepG2 cells treated with lycorine, with the increase of lycorine concentration, clonogenic survival was decreased. When the concentration of lycorine was 2 μM, the clone survival rate of HepG2 (Figure 1C and D) and Huh7 (Figure 1G and H) was zero.

To clarify the impact of lycorine on cell migration, we conducted transwell assays in HepG2 (Figure 1I and J) and Huh7 cells (Figure 1K and L). The results showed that the migrative ability of HCC cells treated with lycorine was obviously decreased. Overall, these data demonstrate that lycorine may inhibit the proliferation and migration of HCC cells *in vitro*.

### Effect of lycorine on cell cycle and apoptosis of HCC cells

**Figure 2:**
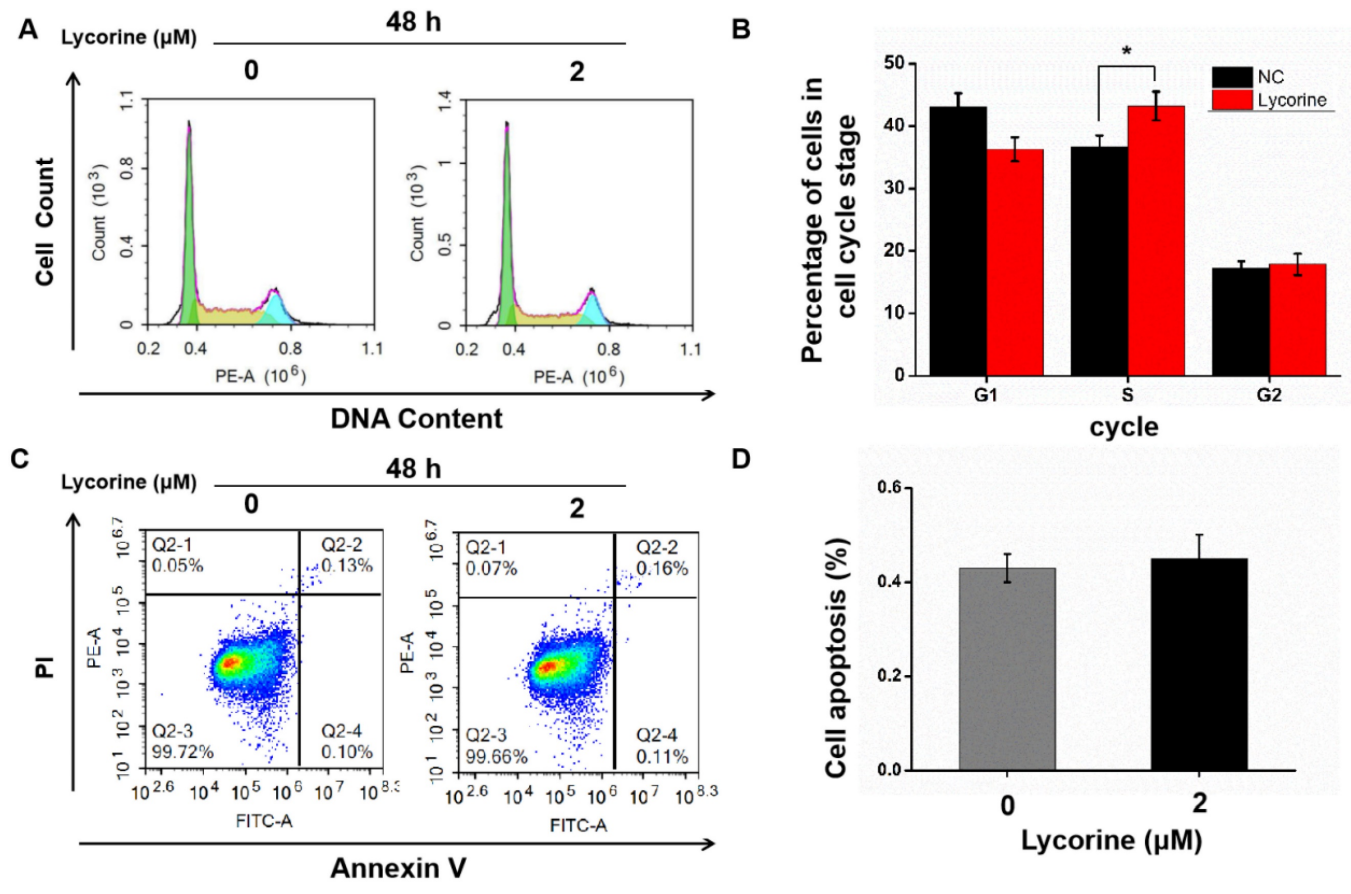
** Effects of lycorine on cell cycle and apoptosis of HepG2 cells.** A and B: Lycorine induced S-phase arrest in HepG2 cells (*P<0.05); C and D: No effect was observed on apoptosis.

**Figure 3:**
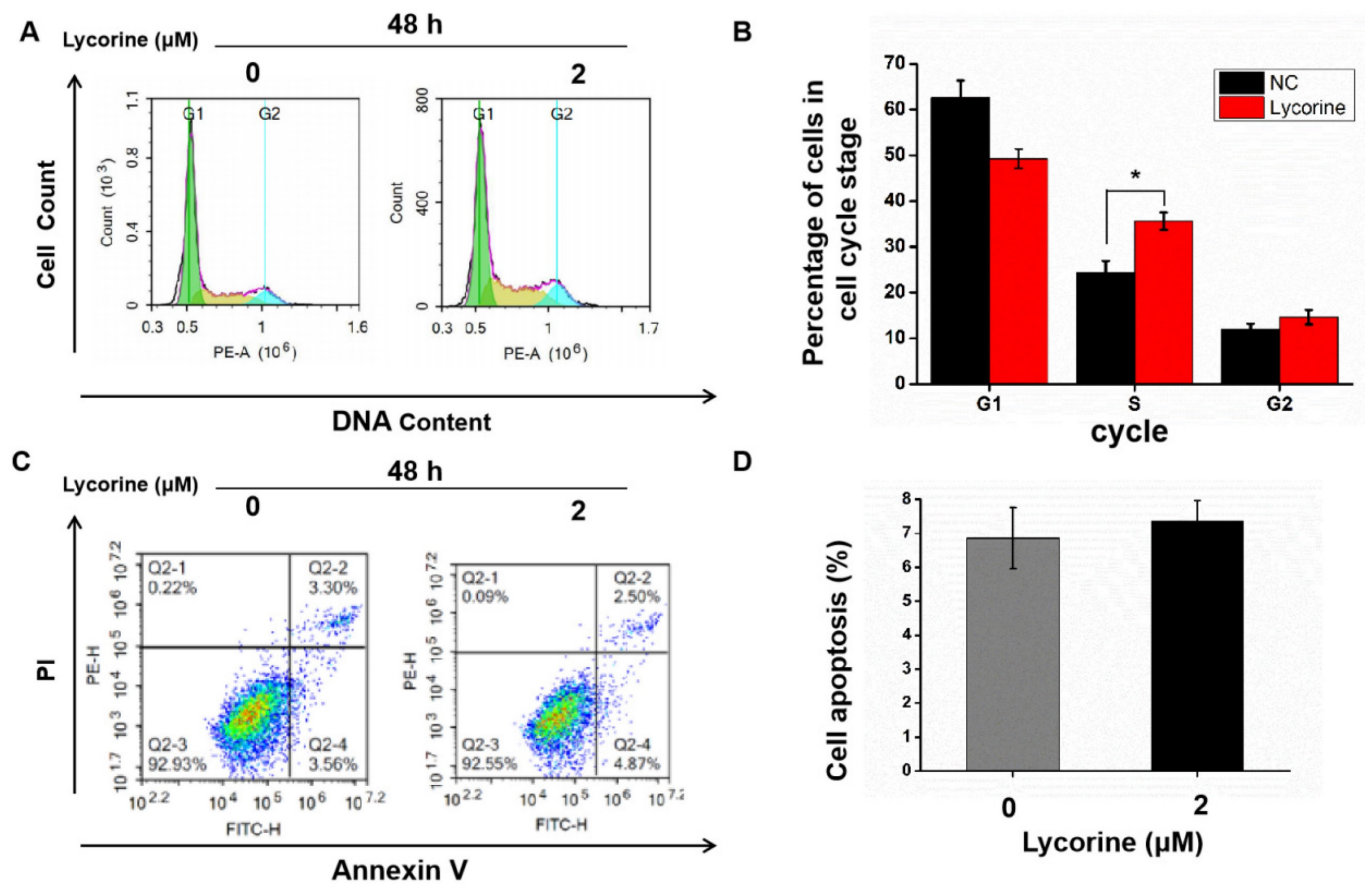
**Effects of lycorine on cell cycle and apoptosis of Huh7 cells.** A and B: Lycorine induced S-phase arrest in Huh7 cells (*P<0.05); C and D: No effect was observed on apoptosis.

Cell cycle analysis using flow cytometry indicated that lycorine induced an arrest of both HepG2 (Figure 2A and B) and Huh7 cells (Figure 2C and D) cells in S-phase, while there was no difference in the numbers of apoptotic cells in HepG2 cells (Figure 3A and B) and in Huh7 cells (Figure 3C and D).

### Lycorine inhibits HCC cell proliferation and migration by down-regulating TKT expression

**Figure 4:**
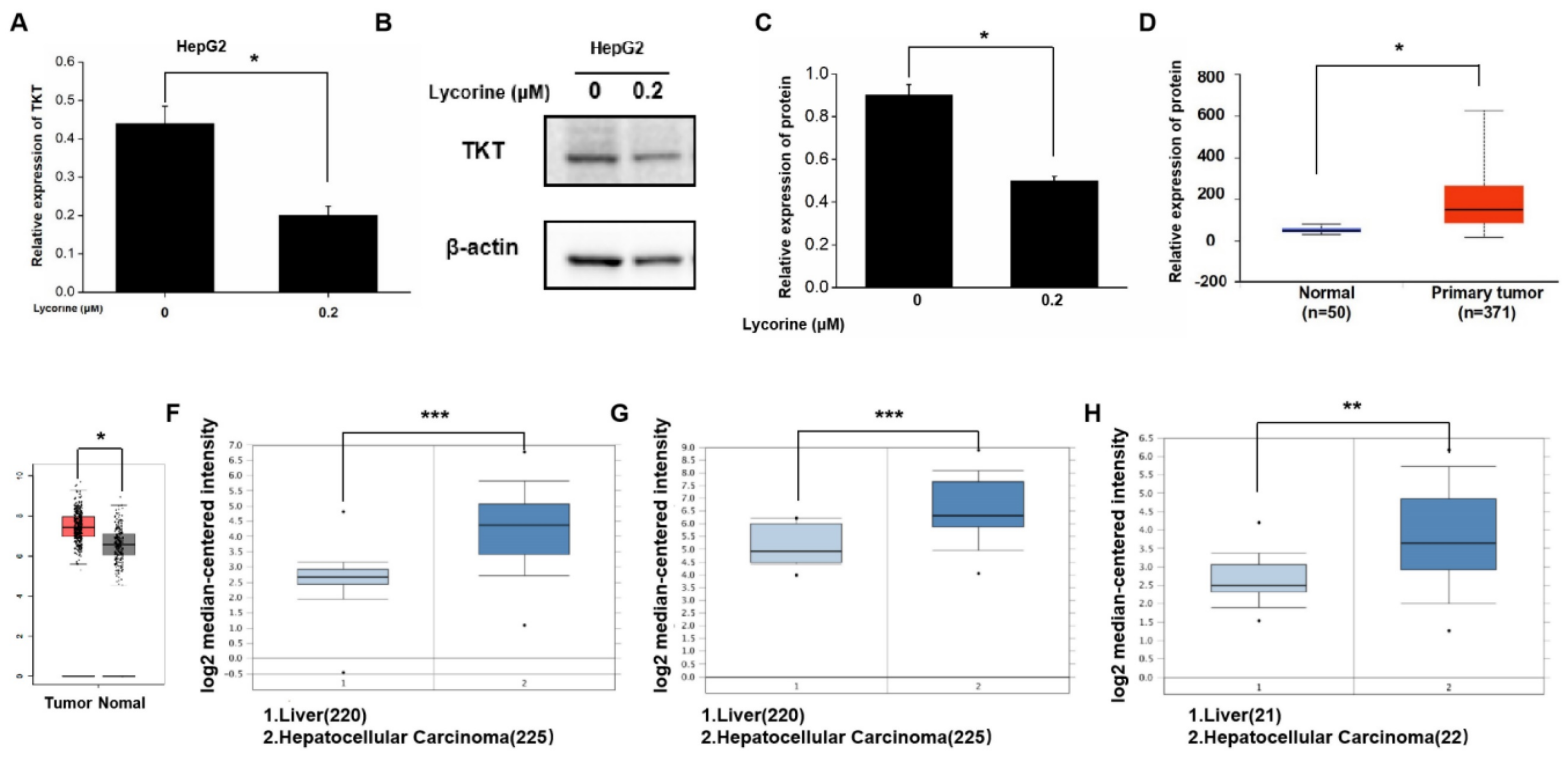
** Lycorine inhibits the expression of transketolase.** A: PCR results showing that the mRNA expression of transketolase (*TKT*) was down-regulated after lycorine treatment in HepG2 cell (*P<0.05); B and C: The protein expression of TKT was down-regulated after lycorine treatment in HepG2 cell as revealed by Western blot analysis (*P<0.05); D-H:The mRNA expression of *TKT* was upregulated in hepatocellular carcinoma tissues compared with normal tissues (*P<0.05*,* **P<0.01, ***P<0.001).

Gene sequencing revealed that 56 genes were down-regulated both in HepG2 and Huh7 cells after lycorine treatment observably, and the expression of *TKT* was down-regulated significantly by 6.8-fold in HepG2 cells and 6.2-fold in Huh7 cells (Table 2). TKT mRNA and protein expression was decreased after lycorine treatment in HepG2 cells as revealed by RT-PCR and Western blot, respectively (Figure 4A-C). We also analyzed the expression of *TKT* mRNA based on online databases and found that the expression *TKT* mRNA was significantly higher in HCC tissues (Figure 4D-H).

### TKT protein expression is significantly increased in HCC tissues

**Figure 5:**
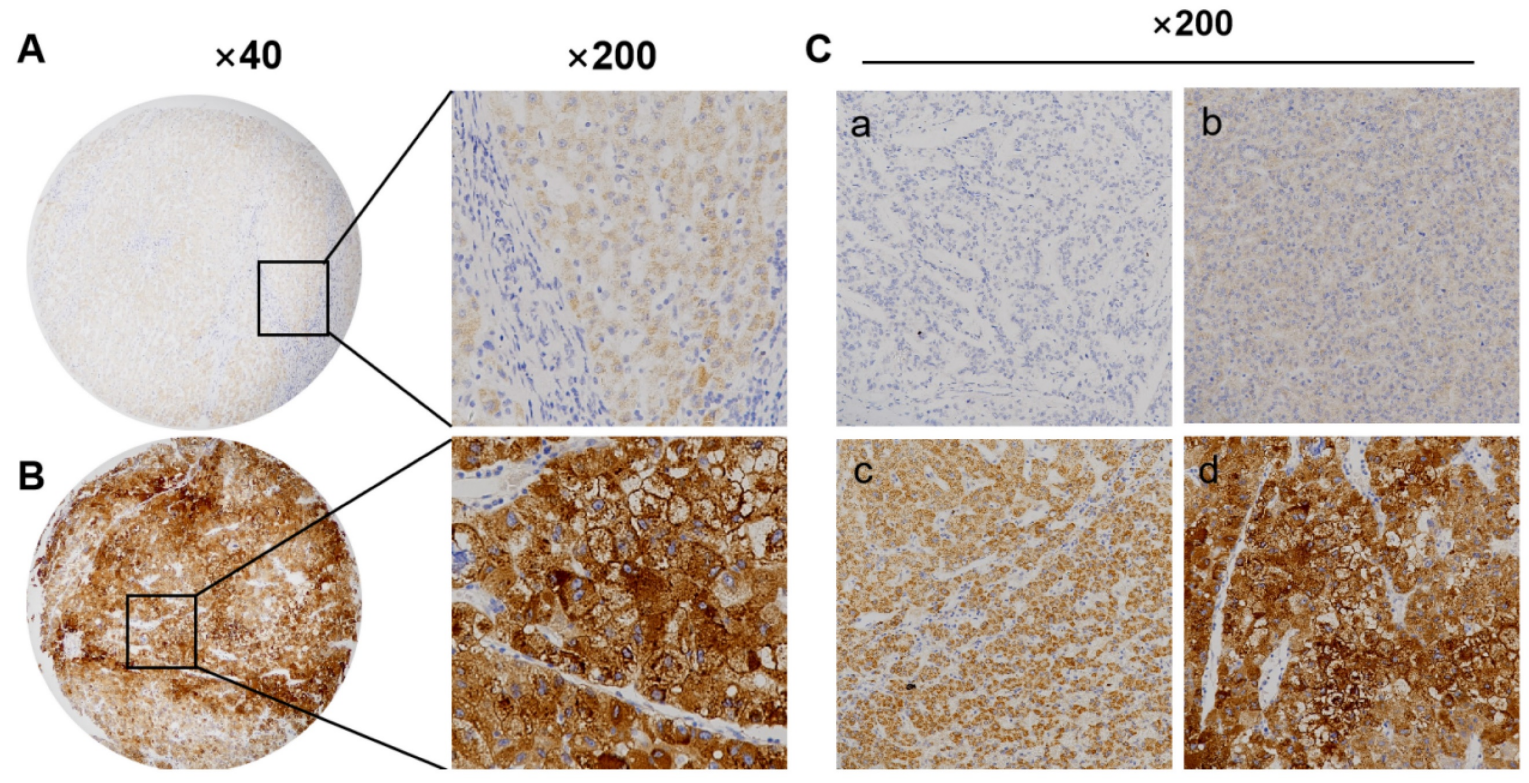
** Transketolase expression significantly increases in hepatocellular carcinoma tissues.** A: Low expression of transketolase (TKT) in tumor tissue; B: High expression of TKT in paired tumor adjacent tissue. Magnification × 200 or × 40; C: (a) Negative (‑), (b) positive (+); (c) positive (++), and (d) positive (+++).

We detect TKT protein expression in tumor and adjacent tissues performed by immunohistochemistry. TKT protein was highly expressed in 54% (108/200) HCC samples, which was situated major in the cytoplasm of cancer, the expression of TKT was also recorded in non-tumor mucosa (Figure 5A-C). The expression of TKT was significantly correlated with tumor size and Edmondson grade, and AFP (*P*<0.01) (Table 1). According to the IHC score, the expression of TKT in tumor was higher than that in non-tumor tissues. There was statistically significant (*P*<0.05).

### Correlation between TKT expression and prognosis

**Figure 6:**
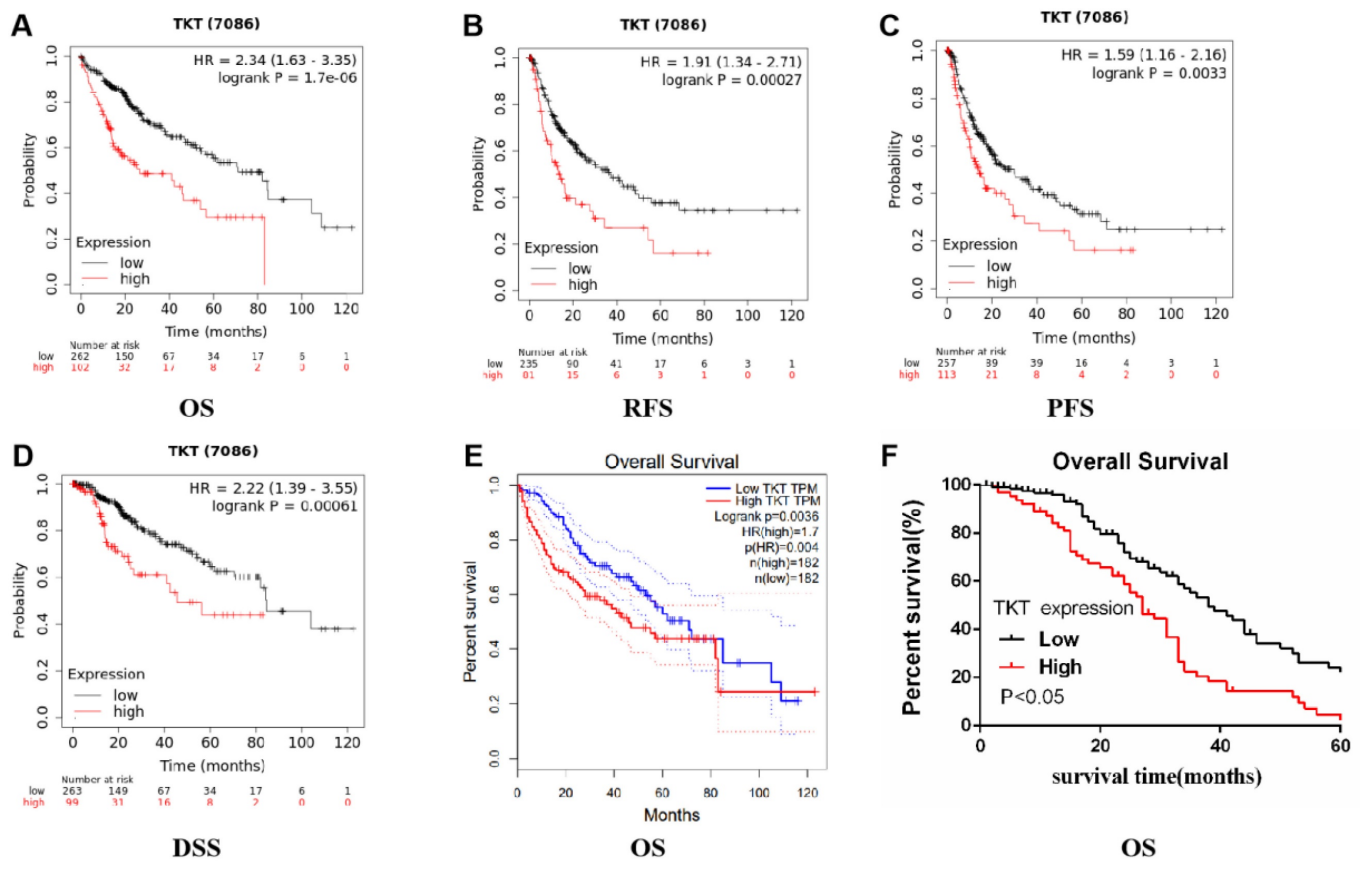
** The expression of transketolase is associated with prognosis in hepatocellular carcinoma.** A-E: Analysis based on Kaplan-Meier plotter and GEPIA databases showed that higher transketolase (TKT) expression was correlated with a poor prognosis in hepatocellular carcinoma; F: Patients with higher TKT expression level were associated with a shorter survival than those with low TKT expression.

We evaluated the prognostic value of TKT expression based on the Kaplan-Meier plotter database. Interestingly, higher TKT expression was shown to correlate with prognosis in HCC (overall survival: HR=2.34, 95%CI=1.63 to 3.35, *P* =1.7e-6; recurrence free survival: HR=1.91, 95%CI=1.34 to 2.71, *P*=0.00027; PFS: HR=1.59, 95%CI=1.16 to 2.16, *P*=0.0033; disease specific survival: HR=2.22, 95%CI=1.39 to 3.55, *P*=0.00061) (Fig 6A-D). Similar results were also obtained based on the TCGA database (overall survival: HR=1.7, *P*=0.004) (Figure 6E). These results indicate that TKT has an effect on the prognosis of HCC.

In our study, the prognostic value of TKT in HCC was estimated by Kaplan-Meier analysis. Patients with high TKT expression had a poor prognosis. The 5-year survival rate of patients with low TKT expression was significantly higher than that of patients with high TKT expression (Figure 6F).

### Potential biological functions of TKT in HCC

**Figure 7:**
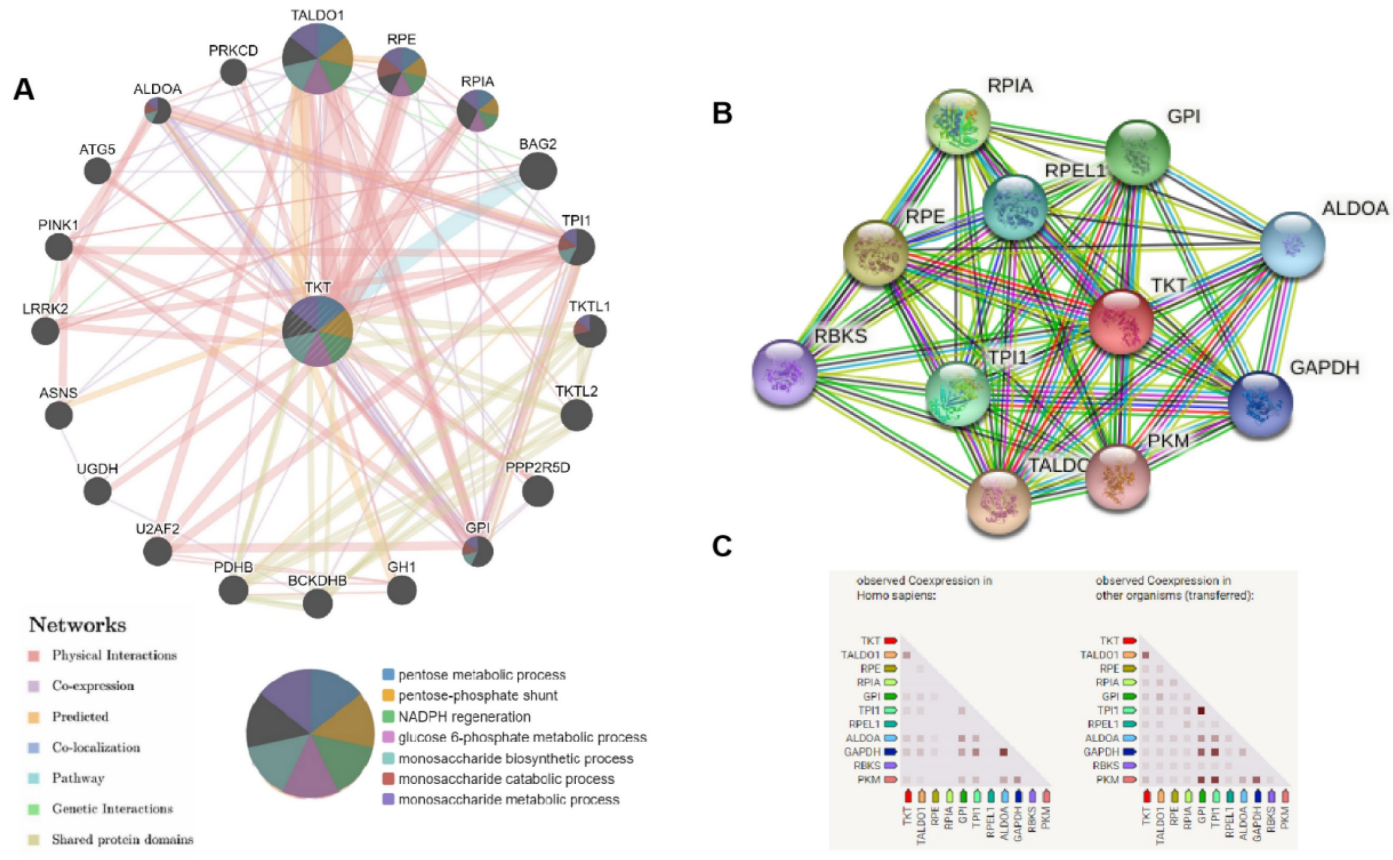
** Potential regulatory network of transketolase in hepatocellular carcinoma analyzed using GeneMANIA and STRING.** A: We analyzed gene-gene functional interaction network of transketolase (TKT) using GeneMANIA, showing the genes with physical interactions, shared signaling path ways, and predicted interactions with TKT; B and C: Protein-protein interaction network of TKT analyzed using STRING. Color images are available online.

In order to investigate the roles of TKT in HCC, we established a gene network with TKT as the core using GeneMANIA (Figure 7A). The results revealed that TALDO1, RPE, RPIA, and BAG2 have shared signaling pathways with TKT, and have interactions with TKT.

We analyzed the interactions of TKT with other partners in HCC using STRING online tools. The results revealed co-expression of TKT with TALDO1 and PGD, which play a key role in regulating a key enzyme of the nonoxidative pentose phosphate pathway (Figure 7B and C).

### Inhibition of lycorine on tumor cells in vivo

**Figure 8:**
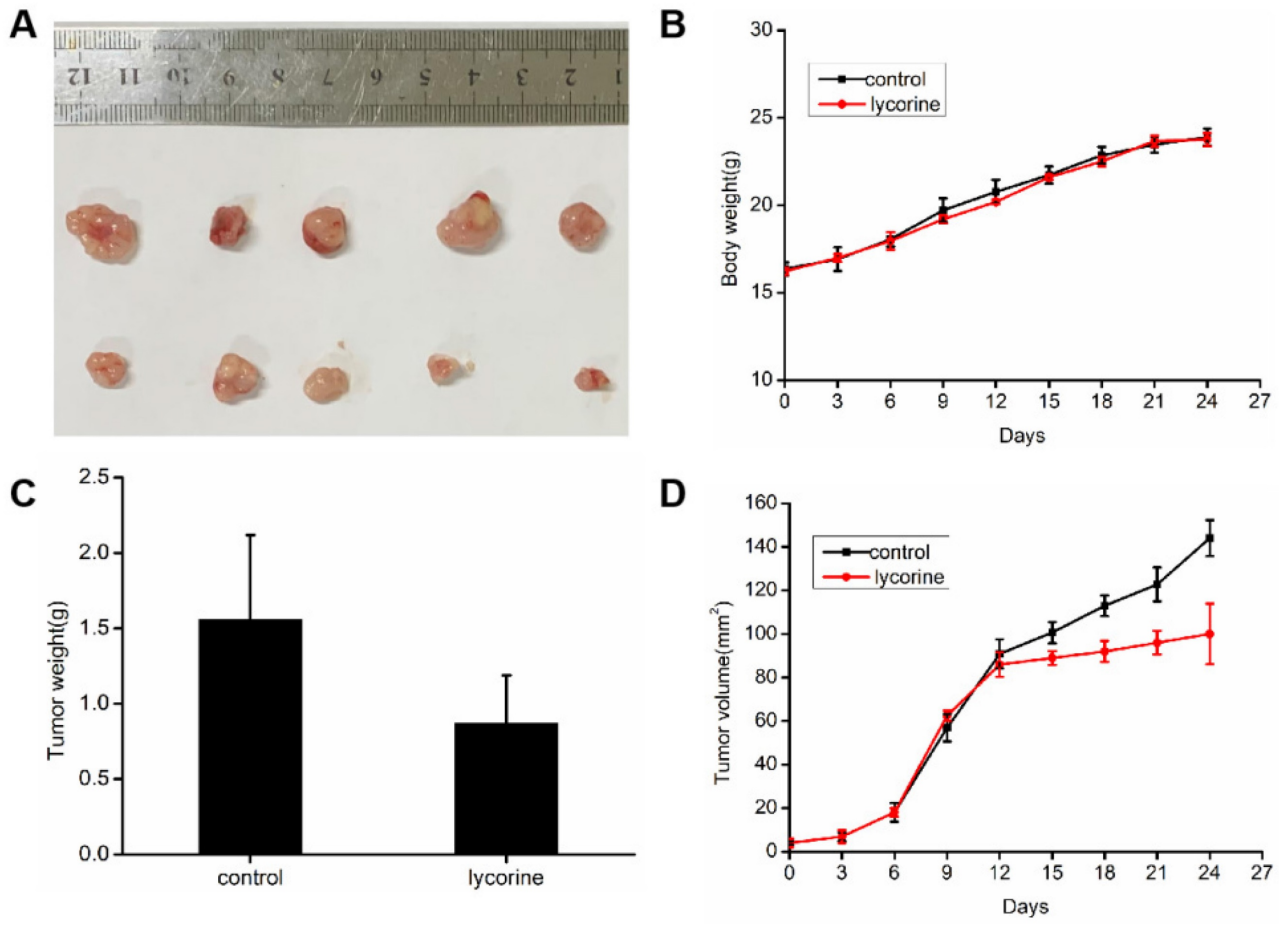
** Inhibition of lycorine on tumor cells in vivo.** A: HepG2 cells were injected subcutaneously into BALB/c mice, and when the average tumor volume reached approximately 100 mm^3^, mice were intraperitoneally injected with PBS or lycorine (10mg/kg/day per mouse) for 15 days. B: The body weights of the mice did not change significantly in the lycorine-treated and control groups. C: The mean tumor weight and volume were reduced in the lycorine-treated group compared with the control group.

To evaluate the inhibitory activity of lycorine against cancer in vivo, we constructed a mouse tumor model by subcutaneous implantation of HepG 2 cells in BALB/c mice. When the volume of tumor reached 100 mm^3^, we treated lycorine and found that the average tumor volume and weight were significantly reduced in the lycorine injection group, but the body weights of the mice did not change significantly (Figure 8A, B, C). The result showed that lycorine has some inhibitory effect on tumor growth and could potentially be a potentially effective therapeutic agent for HCC.

## Discussion

Studies have shown that lycorine has a good anti-cancer effect in liver cancer; it inhibits the proliferation and migration of HepG2 cells by inhibiting ROCK1/cofilin-induced actin^22^, promotes apoptosis and autophagy of HCC cells *via* the TCRP1/AKT/mTOR pathway^9^, and induces mitochondria-dependent apoptosis in HCC cells^23^. We also found that even a low concentration of lycorine significantly inhibited the proliferation, colony formation, and migration of HepG2 cells. However, we discovered that lycorine could block the cell cycle of hepatoma cells at S phase, but had no significant effect on apoptosis. Our study also confirmed that lycorine has an anti-tumor effect. There are many studies reporting on the mechanisms for lycorine to inhibit tumor cell growth and induce apoptosis, which involve target molecules such as MEK2, ROCK1, and miR-186/CDK1; however, it remains unclear which molecule has the main role^9^,^23^,^24^. Thus, we performed gene sequencing of HepG2 cells after lycorine treatment and found that 56 genes in HepG2 cells were down-regulated, of which the *TKT* gene was down-regulated by 6.8-fold and 6.2-fold in HepG2 and Huh7 cells, respectively.

TKT is a thiamine diphosphate-dependent enzyme and elevated TKT activity may enhance the PPP and promote synthesis of 5-phosphoribose and NADPH, leading to nucleotide synthesis, inhibition of oxidative stress, and rapid proliferation of tumor cells. Dasgupta *et al* found that altered cell metabolism and transcriptional program are both markers of cancers to maintain rapid proliferation and metastasis. PFKFB4 up-regulates TKT expression by driving SRC-3 activation and glucose flow to the PPP, thereby providing conditions for rapid division and proliferation, and causing proliferation and metastasis of cancer cells^25^. We found that the proliferative and migratory activities of HepG2 cells were significantly inhibited by a TKT shRNA. Xu *et al* compared the expression of all PPP enzymes in 16 human hepatocarcinoma and corresponding para-cancerous liver tissues by transcriptome sequencing^26^. Most PPP enzymes were significantly up-regulated in human hepatocarcinoma, and TKT was the most abundant and greatly upregulated PPP enzyme in HCC. The results of the TKT activity assay in different states showed that TKT activity was significantly increased in HCC tissues. Intracellular TKT promotes HCC invasion and metastasis through the EGFR pathway^27^. TKT is highly expressed in breast cancer tissues and metastatic lymph nodes, which is associated with a poor prognosis^10^. TKT is also highly expressed in esophageal squamous cell carcinoma, and associated with a poor prognosis^28^. We discovered that the expression of TKT protein was obviously higher in HCC than in para-carcinoma tissues, and its expression was significantly correlated with tumor size and Edmondson grade. Big data analysis using the TCGA database also confirmed that *TKT* mRNA expression is significantly increased in HCC, and its expression correlates with the stage and prognosis of HCC.

In recent years, research has shown that the migration and proliferation of hepatocellular carcinoma cells are related to multiple mechanisms and signaling pathway^29^, invcluding cell cycle arrest, immune infiltration^30^, and post-translational^31^ et al. In future research, we will further explore the specific mechanisms by which lycorine inhibits tumor growth and TKT promotes tumor progression, providing a theoretical basis for its clinical application.

## Conclusions

Lycorine inhibits the proliferation and migration of HCC cells and down-regulates TKT expression. Increased expression of TKT in HCC cells promotes cell invasion and metastasis. Lycorine may regulate reprogramming of glucose metabolism and inhibit the invasion and migration of HCC cells by down-regulating the expression of TKT. However, the mechanism by which lycorine down-regulates TKT and the role of TKT in HCC cell proliferation, invasion, and migration need further research.

## Figures and Tables

**Table 1:** Relationship between TKT expression and pathological parameters of HCC.

Clinical parameters		all cases	TKT		*P* value
			High	Low	
Age (years)					0.927
	<55	92	50	42	
	≥55	108	58	50	
Gender					0.015
	Male	112	52	60	
	Female	88	56	32	
**Tumor Size**					**< 0.001**
	<5	140	64	76	
	≥5	60	44	16	
Tumour number					0.395
	Single	128	72	56	
	multiple	72	36	36	
**Edmondson Grade**					**0.005**
	I+II	136	86	50	
	III	64	22	42	
Metastasis					0.095
	M0	170	96	74	
	M1	30	12	18	
Microvascular invasion					0.295
	Absence	92	46	46	
	Presence	108	62	46	
HBs antigen					0.578
	Negative	56	32	24	
	Positive	144	76	68	
**AFP**					**< 0.001**
	<50	110	32	78	
	≥50	90	76	14	

**Table 2:** Genes with downregulated expression after lycorine treatment.

Gene ID	Gene name	HepG2 Fold change	Huh7 Fold change
7086	TKT	6.834156535	6.206011884
8416	ANXA9	3.5	2.617713853
116535	MRGPRF	2.875	4.119047619
284451	ODF3L2	2.75	3.8
118980	SFXN2	1.125	2.14321608
8811	GALR2	12.2	2.857142857
50943	FOXP3	1.666666667	4.5
50617	ATP6V0A4	9.5	2.5
2542	SLC37A4	9.247311828	2.156761069
113177	IZUMO4	7.875	3.625
80975	TMPRSS5	7.333333333	3.285714286
158787	RIBC1	6.416666667	3.956521739
26751	SH3YL1	6	2.235294118
221527	ZBTB12	5.88034188	2.480519481
79895	ATP8B4	5.5	2.333333333
85366	MYLK2	5.333333333	4.2
2264	FGFR4	5.021505376	2.60420494
144535	CFAP54	5	2.5
64077	LHPP	4.858823529	4.026143791
114757	CYGB	4.666666667	2.205882353
113115	MTFR2	2.062015504	1.406150583
149465	CFAP57	4.5	5
399671	HEATR4	4.5	2.5
55084	SOBP	4.333333333	2.198879552
9315	NREP	4.191011236	2.196969697
102723360	LOC102723360	4.0625	2.34375
150696	PROM2	4	3.5
93659	CGB5	3.956521739	2.368421053
84695	LOXL3	3.837209302	2.142857143
5021	OXTR	3.7	2.681632653
53358	SHC3	3.454545455	4
90557	CCDC74A	3.333333333	2.148648649
644634	LOC644634	3.333333333	3.285714286
643669	CCER2	3.25	3.068965517
445	ASS1	3.122903097	2.364266725
2045	EPHA7	3.090909091	6.15
84083	ZRANB3	3.071428571	2.652173913
2797	GNRH2	3	2.285714286
23563	CHST5	3	5.5
326625	MMAB	2.969230769	2.654480287
256126	SYCE2	2.935779817	2.333333333
84168	ANTXR1	2.753453773	3
387778	SPDYC	2.7	2.875
245812	CNPY4	2.561728395	2.494845361
55005	RMND1	0.668697639	0.737044146
79957	PAQR6	2.524390244	2.289036545
55374	TMCO6	2.435079727	2.201476793
5101	PCDH9	2.4	2.294964029
388436	LOC388436	2.375	2.125
79999	LOC79999	2.375	2.125
26150	RIBC2	2.365771812	3.48
8928	FOXH1	2.272727273	2.259259259
102800317	TPTEP2-CSNK1E	2.265625	4.157894737
79792	GSDMD	2.243523316	2.226012793
51233	DRICH1	2.2	2.133333333
107985388	LOC107985388	2.130434783	2.217391304
